# Neuronal-Activated ILC2s Promote IL-17A Production in Lung γδ T Cells During Sepsis

**DOI:** 10.3389/fimmu.2021.670676

**Published:** 2021-04-30

**Authors:** Weiwei Chen, Dengming Lai, Yuehua Li, Xueke Wang, Yihang Pan, Xiangming Fang, Jie Fan, Qiang Shu

**Affiliations:** ^1^ Department of Thoracic and Cardiovascular Surgery, National Clinical Research Center for Child Health, The Children’s Hospital, Zhejiang University School of Medicine, Hangzhou, China; ^2^ Department of Surgery, University of Pittsburgh School of Medicine, Pittsburgh, PA, United States; ^3^ Research and Development, Veterans Affairs Pittsburgh Healthcare System, Pittsburgh, PA, United States; ^4^ Department of Anesthesiology and Intensive Care Unit, The First Affiliated Hospital, Zhejiang University School of Medicine, Hangzhou, China; ^5^ McGowan Institute for Regenerative Medicine, University of Pittsburgh, Pittsburgh, PA, United States

**Keywords:** group 2 innate lymphoid cells, neuromedin U, sepsis, γδ T cells, IL-17A

## Abstract

**Background:**

Studies have revealed important roles for IL-17A in the development of acute lung injury (ALI) following sepsis. However, the mechanism underlying the regulation of lung IL-17A remains to be fully addressed. Recent studies suggested the effect of neuromedin U (NMU) on immune cell activation and the role of group 2 innate lymphoid cells (ILC2s) in the modulation of IL-17A production. We aimed to gain in-depth insight into the mechanism underlying sepsis-induced lung IL-17A production, particularly, the role of NMU in mediating neuronal regulation of ILC2s and IL-17A-producing γδ T cells activation in sepsis.

**Methods:**

Wild type mice were subjected to cecal ligation and puncture (CLP) to induce sepsis with or without intraperitoneal injection of NMU. The levels of ILC2s, γδ T cells, IL-17A, NMU and NMU receptor 1 (NMUR1) in the lung were then measured. In order to determine the role of NMU signaling in ILC2 activation and the role of ILC2-released IL-9 in ILC2-γδ T cell interaction, ILC2s were sorted, and the genes of *nmur1* and *il9* in the ILC2s were knocked down using CRISPR/Cas9. The genetically manipulated ILC2s were then co-cultured with lung γδ T cells, and the levels of IL-17A from co-culture systems were measured.

**Results:**

In septic mice, the levels of NMU, IL-17A, ILC2s, and IL-17A-producing γδ T cells in the lung are significantly increased, and the expression of NMUR1 in ILC2s is increased as well. Exogenous NMU further augments these increases. The main source of IL-17A in response to CLP is γδ T cells, and lung *nmur1* is specifically expressed in ILC2s. *In vitro* co-culture of ILC2s and γδ T cells leads to increased number of γδ T cells and higher production of IL-17A from γδ T cells, and these alterations are further augmented by septic treatment and exogenous NMU. Genetic knockdown of *nmur1* or *il9* in ILC2s attenuated the upregulation of γδ T cells and IL-17A production.

**Conclusion:**

In sepsis, NMU acting through NMUR1 in lung ILC2s initiates the ILC2 activation, which, in turn, promote IL-17A-producing γδ T cell expansion and secretion of IL-17A. ILC2-derived IL-9 plays an important role in mediating γδ T cell expansion and IL-17A production. This study explores a new mechanism underlying neuronal regulation of innate immunity in sepsis.

## Introduction

Sepsis is the result of the excessive and dysregulated inflammatory response of the body to infection, which often leads to tissue injury, multiple organ dysfunction syndrome (MODS), and death (1). Sepsis-induced mortality is closely associated with secondary acute lung injury (ALI) (2, 3).

Emerging data have shown that IL-17A is an important predictor and therapeutic target in sepsis and secondary ALI. Circulating levels of IL-17A are elevated in human and experimental sepsis (4–6). The study has shown in cecal ligation and puncture (CLP)-induced sepsis mouse model that IL-17A or IL-17A receptor deficiency significantly increased the mortality, which correlated with reduced neutrophil recruitment and more severe bacteremia (7, 8). A study has also shown that early-activated Vγ4δ T cells are the major resource of IL-17A during sepsis and the secretion of IL-17A decreased the mortality of septic mice (9). Furthermore, a recent study using the mouse CLP model demonstrated that IL-17A promoted IgA production, which coupled with a higher survival rate (10). Several types of cells are found to secrete IL-17A including CD4^+^ T helper 17 (Th17) cell, CD8^+^ (Tc17) cell, natural killer T (NKT) cell, γδ T cell, group 3 innate lymphoid cell (ILC3), and “natural” Th17 cell (11, 12). However, in-depth insights into the mechanism underlying the regulation of IL-17A production and secretion in sepsis remains to be fully addressed.

Group 2 innate lymphoid cells (ILC2s) are a special population of cytokine-stimulated and cytokine-producing lymphocytes that exist in mucosal tissues. ILC2s importantly bridge innate and adaptive immunities. Our previous report showed that ILC2s protect lung endothelial cells from pyroptosis in the mouse sepsis model (13). Studies have also found that both ST2^+^ natural ILC2 (nILC2) and ST2^-^ inflammatory ILC2 (iILC2) can produce IL-17A (14–17). However, it remains unclear whether lung ILC2s secrete IL-17A or regulate other cells to secret IL-17A in a setting of sepsis.

It has been reported that the nervous system plays dual roles, either stimulating or suppressing, in the regulation of ILC2 activation in different settings (18–21). For example, studies showed that neuropeptide neuromedin U (NMU) regulates ILC2 activation in asthma and the helminth infection model (18, 19, 22). These findings led us to ask how NMU regulates lung ILC2s in a setting of sepsis and what are the subsequent outcomes in the progression of ALI following sepsis.

In this study, using a mouse sepsis model induced by CLP, we show that NMU acting through NMUR1 on lung ILC2s initiates the ILC2 activation, which, in turn, promotes IL-17A-producing γδ T cell expansion and IL-17A secretion. ILC2-derived IL-9 plays an important role in mediating γδ T cell expansion and IL-17A production. This study explores a new mechanism underlying neuronal regulation of innate immunity in sepsis.

## Methods

### Mice

Male C57BL/6J wild-type mice were purchased from Jackson Laboratories (Bar Harbor, ME). Mice were bred and maintained under specific pathogen-free condition at the Animal Facility of the University of Pittsburgh School of Medicine, VA Pittsburgh Healthcare System, and the Children’s Hospital, Zhejiang University School of Medicine. All mice used in the experiments were 8 weeks old. All mice were performed in compliance with the guidelines of the Institutional Animal Care and Use Committee of the University of Pittsburgh, VA Pittsburgh Healthcare System, and the Children’s Hospital, Zhejiang University School of Medicine, respectively.

### CLP Model and Survival Analysis

Sepsis was induced by CLP procedure as described previously (23). In short, mice were deeply anesthetized with an intraperitoneal injection of xylazine (5 mg/kg) and ketamine (50 mg/kg). A midline incision (1 cm) on the abdomen was performed to allow exteriorization of the cecum. To obtain a moderate CLP, the cecum was ligated 0.8 cm from the apex with 4-0 silk suture and punctured once with a 22-gauge needle in the ligated segment. To induce a severe CLP, the cecum was ligated 1.2 cm from the apex and punctured twice with an 18-gauge needle. A droplet of cecal contents was then slowly squeezed out of the puncture holes. Then the cecum was placed back into the abdomen. The incision was then sutured in two layers. Sham surgery was identical to CLP without puncture and ligation. Mice were fluid resuscitated immediately after surgery (1 ml/mouse sterile saline, subcutaneously). At the specified time point, mice were sacrificed, and lung tissue samples were obtained under sterile condition. Whole blood was collected by cardiac puncture and spun down, and samples were stored at -80°C for further analysis.

In the survival analysis, the survival of animals (5 mice per group) was monitored each 3 hours for 72 consecutive hours after CLP surgery. To relieve pain, mice with signs of imminent death were overdosed with xylazine/ketamine. The survival rate was evaluated, followed by plotting the survival curve.

### Treatment of Mice

In some *in vivo* experiments, mice were injected intraperitoneally (i.p.) with 0.2 µg/g B.W. of NMU-23 peptide (Phoenix Pharmaceuticals, USA) at 6h before and after CLP or with a single dose of NMU-23 peptide (1 µg/g B.W.) at 6h before CLP. At 24h after CLP, lungs were harvested for further analysis. Control mice were treated with PBS.

### Cell Isolation

Cell isolation from lung tissue was performed as described previously (24). Briefly, lungs were perfused with 5 ml cold PBS with 2% heparin through right ventricle of the heart, and then filled with 1 ml HBSS with Liberase ™ (100 μg/ml final concentration) (Roche, USA) and digested in 4 ml HBSS digestion medium for 45 min at 37°C with vortexing every 15 min. The resultant samples were mashed by 70-μm cell strainers, washed with Dulbecco’s modified Eagle media [DMEM; supplemented with 10% fetal bovine serum (FBS) and 1% penicillin/streptomycin (Thermo Fisher Scientific, Pittsburgh, PA, USA)], and treated with RBC Lysis Buffer (eBioscience ™) to lyse red blood cells. Cell suspensions were used for subsequent flow cytometry staining.

### Western Blot

Western blot was performed using standard methods. In short, protein (30 μg) was electropharesed through 12% SDS polyacrylamide gels and transferred to PVDF membranes (Bio-Rad, USA). The membranes were incubated with primary antibodies at 4°C overnight, followed by secondary antibodies tagged with HRP (Thermo Fisher Scientific, USA) at room temperature for 1 hour. The signals were detected using ECL Kit (Pierce Biotech, Rockford, Illinois, USA). A GAPDH antibody was used as a control for whole-cell lysates.

### Flow Cytometry

For flow cytometry analysis, anti-mouse CD16/CD32 antibody (eBioscience, USA) was added to samples at a 1:200 dilution for 20 minutes at 4°C to block nonspecific binding to Fc receptors before cell staining.

LIVE/DEAD Fixable Aqua Dead Cell Stain Kit (eBioscience), Fixable Viability Dye eFluor™ 780 (eBioscience) or 7AAD viability dye (eBioscience) were used to exclude dead cells. Lung cell suspensions were stained with anti-CD45 (30-F11), anti-CD3ϵ (17A2), anti-CD4 (RM4-5), anti-CD5 (53-7.3), anti-CD8α (53-6.7), anti-CD11b (M1/70), anti-CD11c (N418), anti-CD19 (eBio1D3), anti-NK1.1 (PK136), anti-TER119 (TER-119), anti-FceR1α (MAR-1), anti-TCRβ (H57-597), anti-TCRγδ (GL-3), anti-Gr1 (RB6-8C5), anti-Thy1.2 (CD90.2; 53-2.1), anti-CD25 (eBio7D4), anti-CD127 (IL-7Rα; A7R34), anti-IL-17A (eBio17B7) from eBioscience; anti-KLRG1 (2F1), anti-Sca1 (D7) from BD Biosciences, anti-T1/ST2 (DJ8) from MD Biosciences. Lineage was composed by CD3ϵ, CD4, CD5, CD8α, CD11b, CD11c, CD19, NK1.1, TER119, FceR1α, TCRβ, TCR γδ and Gr1; Cell populations were defined as: ILC2s, CD45^+^Lineage^−^Thy1.2^+^T1/ST2^+^CD127^+^CD25^+^KLRG1^+^Sca1^+^; γδ T cells, CD45^+^CD3ϵ^+^TCR γδ^+^CD4^-^TCR β^-^.

For intracellular cytokine protein analysis *ex vivo*, cells were stimulated using the Cell Stimulation Cocktail (eBioscience), containing PMA/Ionomycin/Brefeldin‐A/monensin, for 4 hours at 37°C before staining. Intracellular staining was performed using IC fixation/permeabilization kit (eBioscience).

Flow cytometry analysis and cell sorting were performed using LSR Fortessa, FACS Aria flow cytometers (BD Biosciences) and Cytek Aurora (Cytek Biosciences). The percentage of ILC2s is gated in live CD45^+^Lineage^−^ cells. Data analysis was done using FlowJo software (Tristar).

### Sorting and *In Vitro* Culture of Lung ILC2s and γδ T Cells

For flow cytometric sorting, Lin^-^CD45^+^CD90.2^+^ST2^+^ ILC2s were sorted from the lungs of naive mice by FACSAria (BD Biosciences) or Beckman MoFlo Astrios EQ (Beckman Coulter Life Sciences, Indianapolis, IN, USA). The average purity of ILC2s is > 98%. *In vitro* culture of ILC2s was conducted as previously described (25). Sorted ILC2s were routinely grown in DMEM glutaMAX (Gibco) supplemented with 10% FBS, 100 U/ml penicillin, 100 mg/ml streptomycin, 1% hepes, sodium pyruvate, glutamine at 37°C. Lung ILC2s were plated in 96-well round-bottom plates with two densities (1.5 × 10^4^ or 3.0 × 10^4^ cells/well) in 10 ng/ml rmIL-2 (Biolegend, San Diego, CA, USA), 20 ng/ml rmIL-7 (Biolegend) and 20 ng/ml rmIL-33 (Biolegend) for 6 days. Before use, ILC2s were gently washed twice to remove residual rmIL-2, rmIL-7, and rmIL-33.

Lung γδ T cells were collected after flushing the lungs with 5 ml cold PBS through right ventricle to remove circulating cells. Fresh γδ T cells were enriched from lung by negative and positive selection using the TCRγ/δ^+^ T cell isolation Kit (Miltenyi Biotec, Gladbach Bergische, Germany), then CD45^+^CD3ϵ^+^TCR γδ^+^ γδ T cells were sorted by FACSAria (BD Biosciences) or Beckman MoFlo Astrios EQ (Beckman Coulter Life Sciences, Indianapolis, IN, USA). The average purity of γδ T cells is > 98%. *In vitro* culture of γδ T cells was conducted as previously described (26). Sorted γδ T cells were routinely grown in DMEM glutaMAX (Gibco) supplemented with 10% FBS, 100 U/ml penicillin, 100 mg/ml streptomycin, 1% hepes, sodium pyruvate, glutamine at 37°C. Lung γδ T cells were plated in 96-well round-bottom plates with a density of 5.0 × 10^3^ cells/well, in 100 ng/ml rmIL-1β (Biolegend, San Diego, CA, USA) and 100 ng/ml rmIL-23 (Biolegend) to polarize IL-17A-producing γδ T cells (26–28).

For direct co-culture assays, sorted lung ILC2s were cultured with rmIL-2, rmIL-7 and rmIL-33 for 6 days to obtain mature ILC2s, then sorted lung γδ T cells were added to the each ILC2 well, in DMEM glutaMAX (Gibco) supplemented with 10% FBS, 100 U/ml penicillin, 100 mg/ml streptomycin, 1% hepes, sodium pyruvate, glutamine at 37°C. To maintain ILC2 survival, rmIL-7 (20 ng/ml) was included in all assays with ILC2s as well as controls, including when co-culturing with γδ T cells. ILC2s and γδ T cells were co-cultured for 48 hours before the next tests if not otherwise specified.

The following substances were added to cultures as indicated: NMU (1 or 10 μg/ml, Phoenix Pharmaceuticals), rmIL-1β (100 ng/ml; Biolegend), rmIL-23 (100 ng/ml; Biolegend), LPS (1 μg/ml; Sigma-Aldrich) plus TNF-α (20 ng/ml; Biolegend) were added to mimic sepsis stimulation (13).

### Quantitative Real-Time PCR

Total RNA from sorted cells or tissues were extracted by RNeasy Plus Mini Kit (Qiagen, Germantown, MD, USA) or Trizol method (Thermo Fisher Scientific, Pittsburgh, PA, USA) and stored at -80°C for further analysis. Total RNA concentration was measured using a Nanodrop One spectrophotometer (Thermo Fisher Scientific, Pittsburgh, PA, USA). Total RNA was reverse transcribed using the High Capacity cDNA Reverse Transcription kit (Thermo Fisher Scientific, Pittsburgh, PA, USA) according to the protocol.

Quantitative PCR was conducted in triplicate on a CFX Connect Real-Time PCR Detection System (Bio-Rad, Hercules, CA, USA) with TaqMan Gene Expression Master Mix (Applied Biosystems) using the following TaqMan Gene Expression Assays (Applied Biosystems): *Nmu* (Mm00479868_m1); *Nmur1* (Mm00515885_m1); *Il5* (Mm00439646_m1); *Il9* (Mm00434305_m1); *Il13* (Mm00434204_m1); *Il17a* (Mm00439618_m1).

Gene expression was normalized as n-fold difference to the gene *Hprt1* (Mm00446968_m1) and *S18* (Mm03928990_g1) for mouse according to the cycling threshold. Calculation of mRNA levels was performed with the CFX Manager Software version 3.1 (Bio-Rad).

### Lung Homogenate Assays

For lung homogenate, the whole lung was snap frozen on dry ice homogenized in RIPA buffer (Sigma-Aldrich) containing 0.01% protease and phosphatase inhibitor cocktail (Thermo Scientific). The cell debris and tissue were removed by centrifugation at 19,000 g for 30 min at 4°C. The supernatant was collected for analysis of IL-17A (R&D System) by ELISA according to the manufacturer’s instructions.

### Cytokine Measurements

Blood samples were collected, and plasma was obtained by centrifugation at 5,000 g for 20 min at 4°C. For determination of mouse IL-1β, IL-9, IL-17A and IL-23, Quantikine ELISA Kits from R&D Systems were used according to manufacturers’ instructions.

### Gene Knockdown by CRISPR/Cas9 Technology

For CRISPR/Cas9-mediated gene knockdown, the following synthetic guide RNA (sgRNA) sequences were used: 5’-CGATATGCTGGTGCTCCTGG-3’ (targeting *nmur1*); 5’-GTGAGCGGACAGCTGTGTCA-3’ (targeting *Il5*); 5’-ATTGTACCACACCGTGCTAC-3’ (targeting *Il9*); 5’-CTTCGATTTTGGTATCGGGG-3’ (targeting *Il13*); 5’-AAUGUGAGAUCAGAGUAAU-3’ (non-target control) (ThermoFisher Scientific, USA). *Ex vivo*–expanded ILC2s were transfected with *nmur1/Il5/Il9/Il13* CRISPR/Cas9 plasmid or its non-target control (NTC) in accordance with the manufacturer’s instructions. Transfected cells were cultured for 2 days before next step.

### Statistical Analysis

Statistical analyses were done using GraphPad Prism 7.00 software (GraphPad Software, Inc., La Jolla, CA, USA). Survival differences were assessed using the Kaplan-Meier analysis followed by a log-rank test. Student’s t test or ANOVA was used in all other experiments. Data were expressed as mean ± SEM. A *P* value < 0.05 was considered statistically significant, and significance is presented as * *P* < 0.05, ** *P* < 0.01, *** *P* < 0.001, or **** *P* < 0.0001.

## Results

### Sepsis Induces IL-17A-Producing γδ T Cell Expansion and IL-17A Expression in the Lungs

**Figure 1:**
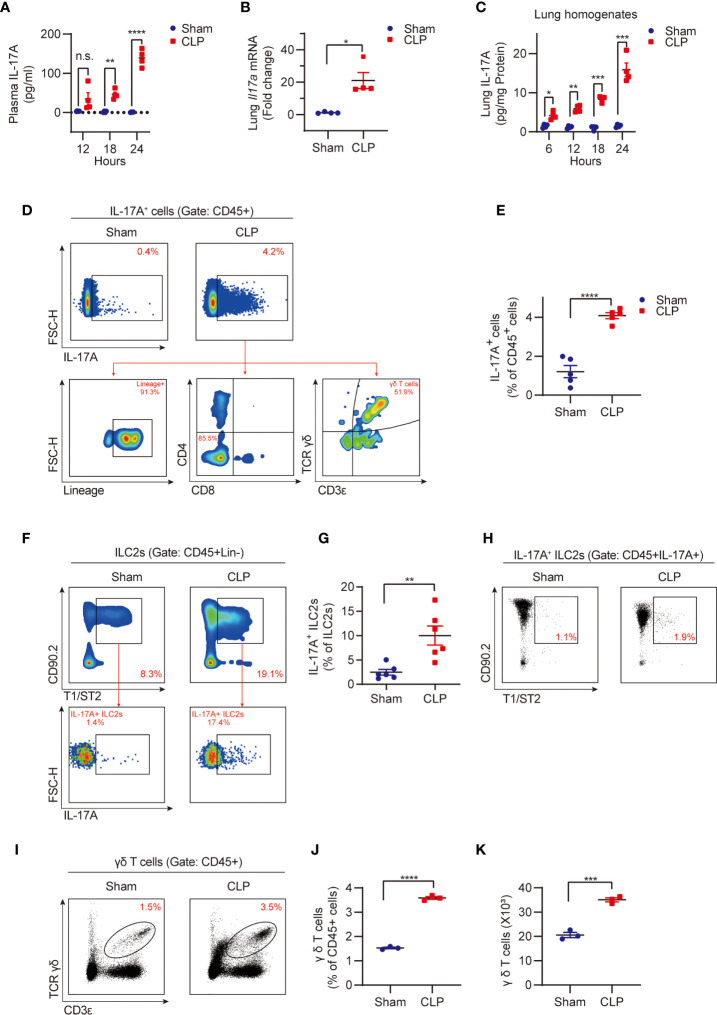
Sepsis induces IL-17A-producing γδ T cell expansion and IL-17A expression in the lungs. Wild type (WT, C57BL/6J) mice were subjected to cecal ligation and puncture (CLP) to induce sepsis or sham surgery, plasma and lung tissue were then collected at different time points as indicated. **(A)** ELISA analysis of plasma IL-17A from CLP or sham mice (n = 4). **(B)** Real-time PCR detection of lung *Il17a* mRNA from mice at 24h after CLP or sham surgery (n = 4). Data were normalized by S18. **(C)** ELISA analysis of IL-17A protein in lung homogenates from CLP or sham mice (n = 4). Data were normalized by protein concentrations. **(D)** Representative flow cytometry plots for IL-17A expression within lung live CD45^+^ populations at 24h after CLP or sham surgery. The relative contribution of different cells (Lineage^-^ILCs, CD4^+^ T cells, CD8^+^ T cells, and γδ T cells) to lung IL-17A^+^ cells was determined. **(E)** The percentages of the IL-17A^+^ cell population within lung live CD45^+^ populations at 24h after CLP or sham surgery (n = 5). **(F)** Representative flow cytometry plots for ILC2 population within lung live CD45^+^Lineage^-^ populations and IL-17A^+^ ILC2 population within ILC2 population at 24h after CLP or sham surgery. **(G)** The percentages of IL-17A^+^ ILC2 population within lung ILC2 population at 24h after CLP or sham surgery (n = 6). **(H)** Representative flow cytometry plots for ILC2 population within lung live CD45^+^IL-17A^+^ populations at 24h after CLP or sham surgery. **(I–K)** Representative flow cytometry plots **(I)**, percentages **(J)**, and numbers **(K)** of γδ T cells within lung live CD45^+^ populations at 24h after CLP or sham surgery (n = 3). All data are mean ± SEM, with symbols representing the values of individual mice. *P < 0.05, **P < 0.01, ***P < 0.001, or ****P < 0.0001, n.s., not significant. One-way ANOVA in **(A, C)**; two-tailed Student’s t-test in **(B, E, G, J, K)**.

Sepsis induced significant increases in plasma IL-17A levels, lung tissue *Il17a* mRNA expression, and IL-17A protein concentration (**Figures 1A–C**), which were consistent with the previous observations (29). Noteworthy, sepsis also induced a markedly increase in the percentage of lung IL-17A-producing cells (**Figures 1D, E**).

Our previous study has shown that sepsis induces ILC2 expansion in the lungs (13). However, it was unknown whether ILC2s secrete IL-17A during sepsis, although it has been reported that in allergic conditions ST2^+^ nILC2s secrete IL-17A (17). We found that IL-17A-producing ILC2s were significantly upregulated in the lungs following the CLP procedure (**Figures 1F, G**). However, importantly, IL-17A-producing ILC2s only occupy 1~2% of total IL-17A-producing cells (**Figure 1H**).

Since multiple cell types, including lineage^-^ ILCs, CD4^+^ T helper 17 (Th17) cells, CD8^+^ (Tc17) cells, and γδ T cells can produce and secret IL-17A (30), we then assessed the relative contribution of these cells to the elevated lung IL-17A in sepsis. Using the flow cytometry gating strategy, we found the γδ T cell lineage, but not lineage^-^ ILCs, CD4^+^ T cells, and CD8^+^ T cells, are the major source of IL-17A (**Figures 1D, H**). This finding underscores the important role of γδ T cells in producing IL-17A in the lung in sepsis. We also found that the percentage and numbers of lung γδ T cells were significantly higher in the CLP group than that in the sham group (**Figures 1I–K**). Collectively, these findings suggest an important role for lung γδ T cells in producing and secretion of IL-17A in sepsis.

### Sepsis Promotes NMU Expressions in the Lung and NMUR1 Expression in Lung ILC2s

**Figure 2:**
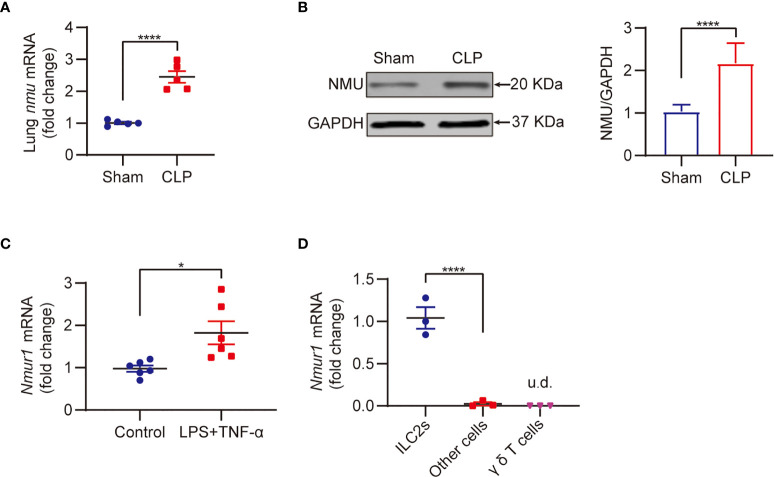
Sepsis promotes NMU expression in the lung and NMUR1 expression in lung ILC2s. **(A, B)** Real-time PCR **(A)** and western blot **(B)** detection of lung NMU expression from CLP or sham mice at 24h (n = 3). **(C)** Real-time PCR detection of nmur1 mRNA in sorted ILC2s under the treatment of LPS + TNF-α for 24h (n = 6). **(D)** Real-time PCR detection of nmur1 mRNA in three cell populations sorted from lung at 24h after CLP surgery (n = 3). All data are mean ± SEM, with symbols representing the values of individual mice. *P < 0.05, ****P < 0.0001, u.d., undetected. One-way ANOVA in **(D)**; two-tailed Student’s t-test in **(A–C)**. Densitometry of western blotting bands was quantified by ImageJ software (gray-scale band analysis) of three independent experiments, non-parametric Mann-Whitney U test.

NMU-NMUR1 signaling has been reported to play an important role in the regulation of inflammation in asthma and helminth infection models (19, 22). To determine the role of NMU-NMUR1 signaling in sepsis-induced inflammation, we measured the expression of NMU in the lungs and NMUR1 expression in ILC2s following CLP. The results show that sepsis markedly increased the expressions of *nmu* mRNA by ~2.4-fold and NMU protein by ~2.1-fold in the lungs (**Figures 2A, B**). We then collected ILC2s by flow sorting and treated the ILC2s with LPS + TNF-α to mimic a septic condition *in vitro*. We found that *nmur1* mRNA expression in the ILC2s treated with LPS + TNF-α is significantly increased as compared to that in the PBS treated (control) group (**Figure 2C**).

Previous studies have shown that lung *nmur1* is selectively expressed in ILC2s (22, 31). To determine this is also true in sepsis, we isolated lung cells from CLP mice, then categorized the cells into three populations, including ILC2s, γδ T cells, and other cells, using flow sorting, followed by measurement of *nmur1* expression in these three populations using real-time qPCR. The results showed that *nmur1* was specifically expressed by ILC2s, but not by γδ T cells and other cells (**Figure 2D**) (22, 31). These findings suggest that ILC2s are the major cell population to respond to the increased NMU expression in the lungs in sepsis.

### NMU Promotes Lung IL-17A-Producing γδ T Cell Expansion

**Figure 3:**
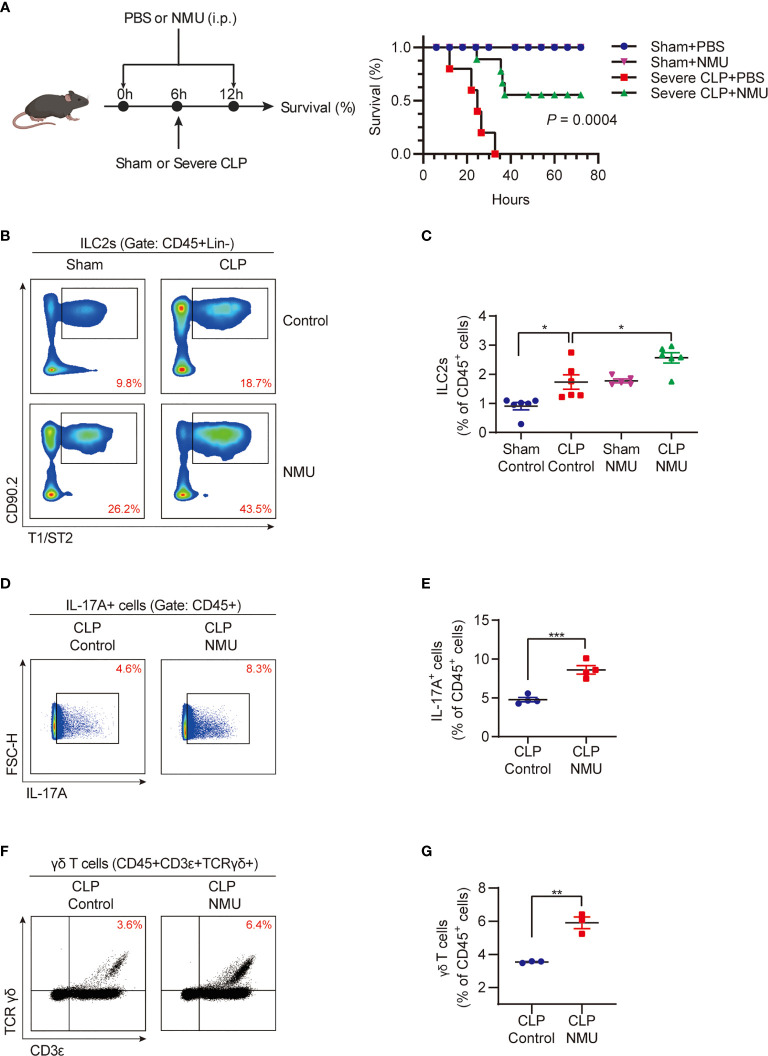
NMU promotes lung IL-17A-producing γδ T cell expansion. **(A)** Survival study of mice monitored for 72h after CLP or sham surgery. Mice received PBS or NMU (0.2 µg/g) at 6h before and after CLP (n = 5). **(B, C) (B)** Representative flow cytometry plots for ILC2 population within lung live CD45^+^Lineage^-^ populations; **(C)** The percentages of ILC2s within lung live CD45^+^ populations at 24h after CLP or sham surgery. Mice received a single dose of NMU (1 µg/g) at 6h before CLP (n = 6). **(D, E)** Representative flow cytometry plots **(D)** and percentages **(E)** of the IL-17A^+^ cell population within lung live CD45^+^ populations at 24h after CLP or sham surgery. Mice received a single dose of NMU (1 µg/g) at 6h before CLP (n = 4). **(F, G)** Representative flow cytometry plots **(F)** and percentages **(G)** of γδ T cell population within lung live CD45^+^ populations at 24h after CLP or sham surgery. Mice received a single dose of NMU (1 µg/g) at 6h before CLP (n = 4). All data are mean ± SEM, with symbols representing the values of individual mice. *P < 0.05, **P < 0.01, ***P < 0.001. Kaplan–Meier analysis in **(A)** One-way ANOVA in **(C)** two-tailed Student’s t-test in **(E, G)**.

Based on the data shown above, we hypothesized that NMU might act through ILC2s to upregulate lung IL-17A-producing γδ T cells. To test this hypothesis, we intraperitoneally injected (i.p.) recombinant NMU into the CLP mice at 6 h before and 6 h after the CLP procedure (**Figure 3A**). We found that NMU significantly reduced the mortality of septic mice (**Figure 3A**). Furthermore, exogenous NMU increased the percentage of ILC2s (**Figures 3B, C**), IL-17A-producing cells (**Figures 3D, E**), and γδ T cells (**Figures 3F, G**) in the lungs of septic mice; whereas NMU administration did not alter the percentage of IL-17A^+^ ILC2s (**Supplemental Figures A, B**). Given that *nmur1* is specifically expressed by ILC2s but not by γδ T cells, the increased γδ T cells in response to NMU is mediated through ILC2s.

### ILC2s Mediate NMU-Induced Increase in Lung γδ T Cells

**Figure 4:**
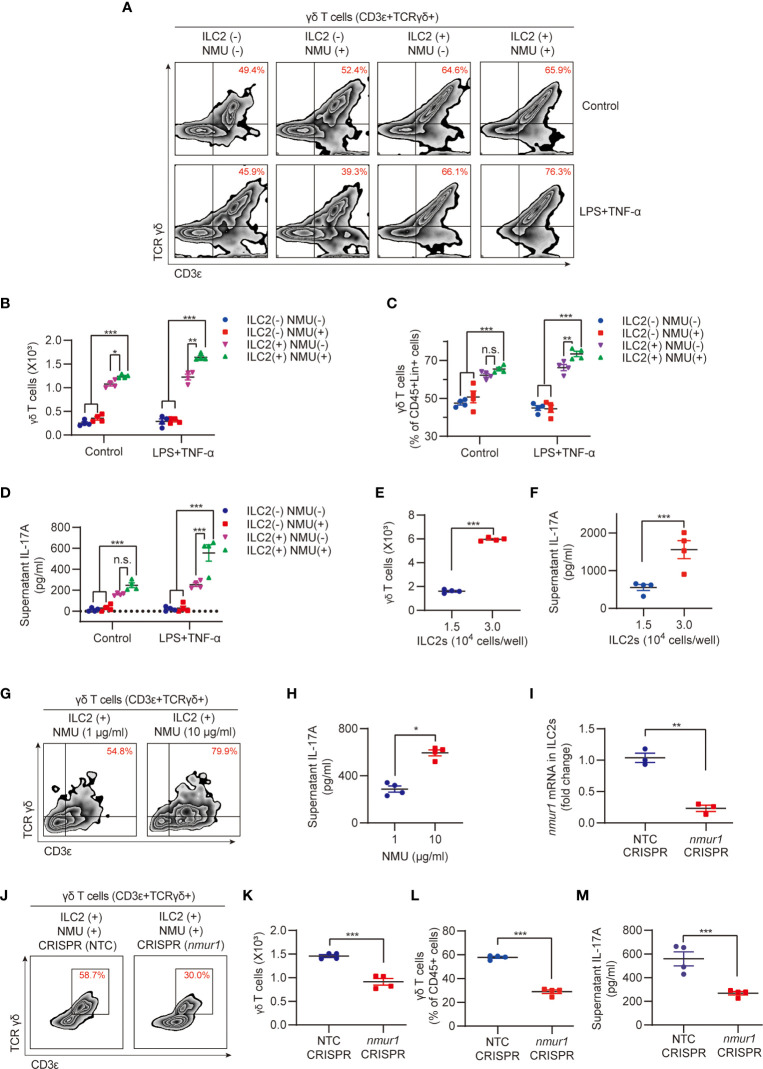
ILC2s mediate NMU-indued increase in lung γδ T cells. **(A–D)** Representative flow cytometry plots **(A)**, numbers **(B)**, percentages **(C)** of γδ T cell population, and ELISA analysis **(D)** of supernatant IL-17A in different groups. ILC2s and γδ T cells were co-cultured for 48h with or without NMU (10 μg/ml). IL-1β (100 ng/ml) and IL-23 (100 ng/ml) were added to polarize IL-17A-producing γδ T cells, LPS (1 μg/ml) plus TNF-α (20 ng/ml) were added to mimic sepsis stimulation (n = 4). **(E, F)** Numbers **(E)** of γδ T cell population and ELISA analysis **(F)** of supernatant IL-17A in groups co-cultured with different numbers of ILC2s. ILC2s and γδ T cells were co-cultured for 48 hours with NMU (10 μg/ml) (n = 4). **(G, H)** Representative flow cytometry plots **(G)** of γδ T cell population and ELISA analysis **(H)** of supernatant IL-17A in co-culture group with different concentrations of NMU (1 or 10 μg/ml). ILC2s and γδ T cells were co-cultured for 48h (n = 4). **(I)** Real-time PCR detection of *nmur1* mRNA in ILC2s after *nmur1* sgRNA transfection using CRISPR/Cas9 approach for 48h (n = 3). **(J–M)** Representative flow cytometry plots **(J)**, numbers **(K)**, percentages **(L)** of γδ T cell population, and ELISA analysis **(M)** of supernatant IL-17A in control and *nmur1* knockdown groups. ILC2s and γδ T cells were co-cultured for 48h (n = 4). All data are mean ± SEM, with symbols representing the values of individual mice. *P < 0.05, **P < 0.01, ***P < 0.001, n.s., not significant. One-way ANOVA in **(B–D)**; two-tailed Student’s t-test in **(E, F, H, I, K–M)**.

To determine the role of ILC2s in mediating NMU-induced upregulation of lung γδ T cells, we applied an *in vitro* ILC2s and γδ T cells coculture system. IL-17A was undetectable in the culture supernatant of the ILC2 alone group after NMU treatment (**Supplemental Figure C**). However, coculture of ILC2s and γδ T cells with the treatment of NMU resulted in significant increases in the number and percentage of γδ T cells (**Figures 4A–C**) and supernatant IL-17A concentrations (**Figure 4D**). NMU failed to induce γδ T cell expansion and IL-17A release in γδ T cell alone group (**Figures 4A–D**). More importantly, the supernatant IL-17A concentrations were further elevated in the ILC2-γδ T cell coculture group treated with NMU and LPS + TNF-α (**Figure 4D**).

To further establish the role of ILC2s in regulating γδ T cell expansion and IL-17A producing, we cocultured γδ T cells (5.0 × 10^3^ cells) with different numbers of ILC2s (1.5 × 10^4^ and 3.0 × 10^4^ cells/well). After 48-hour coculture, the final numbers of γδ T cells in the group co-cultured with 3.0 × 10^4^ ILC2s/well were ~3.2-fold higher than that in the group cocultured with 1.5 × 10^4^ ILC2s/well (**Figure 4E**); and the supernatant IL-17A concentrations of 3.0 × 10^4^ ILC2s/well group were also significantly higher than that of 1.5 × 10^4^ ILC2s/well group (**Figure 4F**). In addition, we treated the cocultures with different NMU concentrations (1 μg/ml and 10 μg/ml) but fixed the number of ILC2s **(**1.5 × 10^4^ cells/well). We found that higher NMU concentration (10 μg/ml) induced higher numbers of γδ T cells and higher IL-17A levels as compared to the group treated with lower NMU concentration (1 μg/ml) (**Figures 4G, H**). These results further suggested that ILC2s mediate NMU-induced γδ T cell expansion and IL-17A production.

To confirm the role of NMUR1 in ILC2s in transducing NMU signaling, we knocked down *nmur1* in ILC2s using the CRISPR/Cas9 approach, which was confirmed by RT-PCR, as shown in **Figure 4I**. Knockdown of *nmur1* significantly attenuated γδ T cell expansion in response to NMU in the co-culture system (**Figures 4J–L**), and decreased IL-17A production (**Figure 4M**).

### IL-9 Mediates ILC2 Regulation of γδ T Cell Expansion and IL-17A Production

Next, we wanted to identify the possible mediators that mediate the ILC2 regulation of γδ T cells in the co-culture system. Previous studies have shown that IL-1β and IL-23 can polarize γδ T cells to produce IL-17A (26–28, 32). Thus, we measured the supernatant levels of IL-1β and IL-23 and found that the concentrations of both cytokines were not changed in all groups of ILC2 cultures (**Supplemental Figures D, E**).

**Figure 5:**
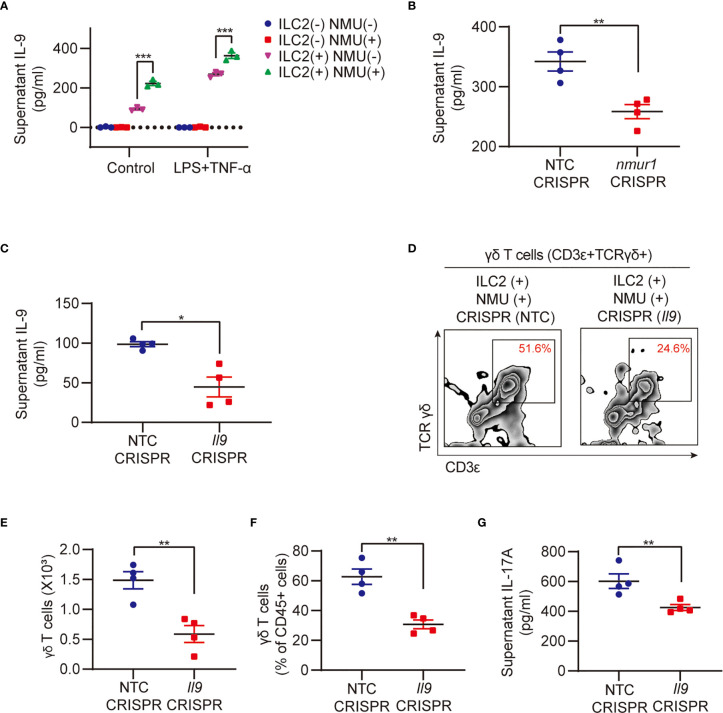
IL-9 mediates ILC2 regulation of γδ T cell expansion and IL-17A production. **(A)** ELISA analysis of supernatant IL-9 in different groups (n = 3). ILC2s and γδ T cells were co-cultured for 48h with or without NMU (10 μg/ml). **(B)** ELISA analysis of supernatant IL-9 in groups after *nmur1* sgRNA transfection using CRISPR/Cas9 approach for 48h and then co-cultured with γδ T cells for 48h (n = 4). **(C)** ELISA analysis of supernatant IL-9 in groups after *Il9* sgRNA transfection using CRISPR/Cas9 approach for 48h (n = 4). **(D–G)** Representative flow cytometry plots **(D)**, numbers **(E)**, percentages **(F)** of γδ T cell population, and ELISA analysis **(G)** of supernatant IL-17A in control and *Il9* knockdown groups. ILC2s and γδ T cells were co-cultured for 48h (n = 4). All data are mean ± SEM, with symbols representing the values of individual mice. *P < 0.05, **P < 0.01, ***P < 0.001. One-way ANOVA in **(A)**; two-tailed Student’s t-test in **(B, C, E, F, G)**.

Our previous studies have shown that ILC2-derived IL-9 serves as an important mediator in the interaction between ILC2s and lung endothelial cells (13). Reports also showed that NMU can induce ILC2s to secrete IL-9 (18, 22), and IL-9/IL-9R signaling regulates γδ T-cell activation (33). In our current coculture experiments of ILC2 with γδ T cells, we observed a significant increase in supernatant IL-9 in response to treatment with NMU and LPS + TNF-α (**Figure 5A**). We further found that in cocultures of *nmur1* knockdown ILC2s with γδ T cells, supernatant IL-9 levels were remarkably lower than that in cocultures of WT ILC2 with γδ T cells (**Figure 5B**). In order to further determine the role of IL-9 in mediating γδ T cell activation, we knocked down IL-9 in ILC2s using a CRISPR/Cas9 approach. The efficiency of *Il9* knockdown was confirmed by detecting culture supernatant IL-9 using ELISA (**Figure 5C**). Coculture of *Il9* knockdown ILC2s with γδ T cells resulted in lower γδ T cell expansion (**Figures 5D–F**) and lower levels of supernatant IL-17A (**Figure 5G**).

To determine if other ILC2-derived cytokines are also involved in mediating the interaction between ILC2s and γδ T cells, we knocked-down IL-5 and IL-13 in ILC2s using the CRISPR/Cas9 method, respectively. We found that the knockdown of IL-5 and IL-13 in ILC2s did not affect γδ T cell expansion and IL-17A production in the co-culture system (data not shown).

Collectively, the data demonstrate an important role for IL-9 in mediating ILC2 regulation of γδ T cell expansion, activation, and subsequent production of IL-17A.

## Discussion

Emerging data suggested the important role of IL-17A in the regulation of inflammation (4–6, 34–36). Although most of the reports have shown that IL-17A plays a beneficial role in improving inflammation (7–10, 35, 36), several studies demonstrated detrimental effects of IL-17A in the development of inflammation (37–40). This ambiguity in the current literature, coupled with the fact that studies on the mechanism of regulation of IL-17A production in sepsis-induced lung injury are lacking, highlights the need for further elucidating how is lung IL-17A regulated and what is the role for IL-17A in the development of ALI and systemic inflammation following sepsis. In this study, we demonstrate that sepsis-induced NMU acting through NMUR1 on lung ILC2s initiates the ILC2 activation, which, in turn, promotes IL-17A-producing γδ T cell expansion and secretion of IL-17A. ILC2-derived IL-9 plays an important role in mediating γδ T cell expansion and IL-17A production.

ILC2s play an important role in bridging innate and adaptive immunities and are functionally similar to polarized Th2 cells (41). ILC2s serve as a potent player in maintaining mucosal homeostasis and host defense against infection in the septic lung (41–43). Regulation of ILC2 activation is multifaceted (44). Recently, the neuronal regulation of ILC2s has been reported (41). Various neuropeptides such as substance P, VIP, CGRP, NMU, and NMB were found to modulate ILC2s. Yet, the mechanism underlying neuronal regulation of ILC2s in sepsis remains unclear. NMU is mainly released by cholinergic sensory neurons originating from the dorsal root ganglion (DRG), but not parasympathetic neurons in the vagal ganglion (41, 45). NMU is also occasionally secreted by some antigen-presenting cells, including monocytes, B cells, and dendritic cells (46). Thus, it is suggested to play an important role in the regulation of adaptive and innate immunity. Recent studies reported that NMU from lamina propria plays a regulatory role in mice type 2 innate immunity through binding to the Nmur1, which is selectively enriched in ILC2s, and NMU-expressing neurons are close vicinity to ILC2s in the lungs (18, 19, 22). In a mice model of worm infection in the lungs and intestine, stimulation of ILC2s with NMU led to strong and immediate production of tissue protection and innate inflammatory cytokines in an NMUR1-dependent manner, thereby alleviating worm burden (18). The report also showed that NMU-activated ILC2s increase the number of lung eosinophils and mast cells, thus alleviating antihelminth responses (18, 19, 22). In this study, we discovered that the lung expression of NMU is elevated during sepsis, and NMU receptor NMUR1 is selectively expressed in the lung ILC2s. This finding suggests an important role for ILC2s as an executive cell population to mediate NMU-regulated downstream events in the lung during sepsis. Indeed, we found in our current study that NMU-induced γδ T cell expansion, activation, and IL-17A production requires ILC2s in the coculture system, and *numr1* deletion in ILC2s disabled NMU-induced γδ T cell activation.

Unlike conventional αβ T cells, γδ T cells are special T cells that exhibit distinctive antigen recognition patterns different from those of αβ T cells and have different functional subsets, defined by the several usages of Vδ and Vγ gene repertoire (47, 48). Antigen processing is not required for γδ T cells to recognize an infection, since γδ T cells can quickly react to various antigens *via* innate surface receptors (49–52) and secret high levels of IL-17A and IFN-γ, both are signature cytokines of γδ T cells (53–57). γδ T cells are a major innate source of IL-17A in the mouse and occupy mostly barrier surfaces, such as the skin and mucosa, as well as secondary lymphoid organs (58, 59). γδ T cells play critical roles in the regulation of inflammation in mouse sepsis model (36, 60–64). The accumulation of γδ T cells in the lungs of CLP mice associates beneficial outcomes of septic mice (60, 61). The protective functions of γδ T cells during experimental sepsis have been attributed to the production of IL-17A, which improves bacterial clearance and triggers neutrophil recruitment (36, 65–67).

ILC2s and γδ T cells share several similarities. γδ T cells are also considered as a bridge linking innate and adaptive immune systems. A recent study showed that tissue-resident lung ILC2s have *TCRγ* gene rearrangements similar to γδ T cells under steady-state conditions. Rearranged *TCRγ* gene in ILC2s is nonfunctional and aberrant, and thus, it is suggested that ILC2s may arise from failed γδ T cell development (68). Given the similarities between ILC2s and γδ T cells in the immune system, we aimed to gain an insight into the interaction between ILC2s and γδ T cells, particularly, pertaining to the precise regulation of lung IL-17A production in sepsis, as the source of IL-17A is controversial. The data from the current study showed that ILC2s can secret IL-17A. However, IL-17A-producing ILC2s only occupy ~2% of total IL-17A-producing cells. ILC2s are not the major source of IL-17A in the lung in sepsis. Our results then showed that ILC2s increase the number of IL-17A-producing γδ T cells, which associate with increased IL-17A secretion. These ILC2s-induced increases can be further exacerbated by NMU and LPS + TNF-α septic treatment. These results establish a determinate role for ILC2s in upregulation of γδ T cell expansion and production of IL-17A in the lung in sepsis.

Recently, IL-9 has been reported to be involved, either beneficially or deleteriously, in the pathogenesis of some diseases related to inflammation (69, 70). ILC2 is the main source of IL-9 in mouse lung tissue in physiological or inflammatory circumstances (71). Our data showed that the knockdown of *Il9* in ILC2s decreases the number of IL-17A-producing γδ T cells, which associates with decreased IL-17A secretion, in response to NUM and LPS + TNF-α. These findings strongly suggest a role for IL-9 in mediating the ILC2 regulation of IL-17A-producing γδ T cell expansion and secretion of IL-17A.

In summary, this study shows that NMU acting through NMUR1 on lung ILC2s initiates the ILC2 activation, which, in turn, promotes IL-17A-producing γδ T cell expansion and IL-17A secretion. ILC2-derived IL-9 plays an important role in mediating γδ T cell expansion and IL-17A production. This study explores a new mechanism underlying neuronal regulation of innate immunity in sepsis.

## Data Availability Statement

The raw data supporting the conclusions of this article will be made available by the authors, without undue reservation.

## Ethics Statement

The animal study was reviewed and approved by Institutional Animal Care and Use Committee of the University of Pittsburgh, VA Pittsburgh Healthcare System Institutional Animal Care and Use Committee of the Children’s Hospital, Zhejiang University School of Medicine.

## Author Contributions

WC conducted the experiments, collected the data, performed the data analysis, and drafted the manuscript. WC, XF, JF, and QS conceived and designed the study. DL, YL, XW, and YP selected and collected the samples. XF, JF, and QS reviewed and finalized the manuscript. All authors contributed to the article and approved the submitted version.

## Funding

This work was supported by the Zhejiang Provincial Program for the Cultivation of High-level Innovative Health talents 2016-6 (QS), the National Natural Science Foundation of China 81671956 (QS), the National Natural Science Foundation of China 81901989 (DL), China Postdoctoral Science Foundation 2019M652108 (DL), Natural Science Foundation of Zhejiang Province LY21H150005 (DL). This work was supported by the VA Merit Awards 1I01BX002729 (JF) and 1I01 BX004838 (JF), and VA BLR&D Award 1IK6BX004211 (JF). WC was supported in part by a two-year China Scholarship Council scholarship 201806320166. The funders had no role in study design, data collection and analysis, decision to publish, or preparation of the manuscript.

## Conflict of Interest

The authors declare that the research was conducted in the absence of any commercial or financial relationships that could be construed as a potential conflict of interest.

## Supplementary Material

Supplementary Figure 
**(A)** Representative flow cytometry plots for IL-17A^+^ILC2 population within lung live CD45^+^Lineage^-^CD90.2^+^ populations at 24h after CLP. **(B)** The percentages of IL-17A^+^ ILC2 population within lung ILC2 population at 24h after CLP (n = 6). **(C)** ELISA analysis of supernatant IL-17A in ILC2 alone group. ILC2s were treated with NMU (10 µg/ml) (n = 3). **(D, E)** ELISA analysis of supernatant IL-1β **(D)** and IL-23 **(E)** in different groups. ILC2s and γδ T cells were co-cultured for 48h with or without NMU (10 μg/ml). IL-1β (100 ng/ml) and IL-23 (100 ng/ml) were added to polarize IL-17A-producing γδ T cells, LPS (1 μg/ml) plus TNF-α (20 ng/ml) were added to mimic sepsis stimulation (n = 3). All data are mean ± SEM. n.s., not significant, u.d., undetected. One-way ANOVA in **(D, E)**; two-tailed Student’s t-test in **(B)**.

## References

[1] Cecconi M Evans L Levy M Rhodes A. Sepsis and Septic Shock. Lancet (2018) 392(10141):75–87. 10.1016/S0140-6736(18)30696-2 29937192

[2] Fisher BJ Kraskauskas D Martin EJ Farkas D Wegelin JA Brophy D. Mechanisms of Attenuation of Abdominal Sepsis Induced Acute Lung Injury by Ascorbic Acid. Am J Physiol Lung Cell Mol Physiol (2012) 303(1):L20–32. 10.1152/ajplung.00300.2011 22523283

[3] Santacruz CA Pereira AJ Celis E Vincent JL. Which Multicenter Randomized Controlled Trials in Critical Care Medicine Have Shown Reduced Mortality? A Systematic Review Crit Care Med (2019) 47(12):1680–91. 10.1097/CCM.0000000000004000 31567349

[4] Li Y Wei C Xu H Jia J Wei Z Guo R. The Immunoregulation of Th17 in Host Against Intracellular Bacterial Infection. Mediators Inflammation (2018) 2018:6587296. 10.1155/2018/6587296 PMC5884031 29743811

[5] Chamoun MN Blumenthal A Sullivan MJ Schembri MA Ulett GC. Bacterial Pathogenesis and interleukin-17: Interconnecting Mechanisms of Immune Regulation, Host Genetics, and Microbial Virulence That Influence Severity of Infection. Crit Rev Microbiol (2018) 44(4):465–86. 10.1080/1040841X.2018.1426556 29345518

[6] Gouda MM Bhandary YP. Acute Lung Injury: IL-17A-Mediated Inflammatory Pathway and Its Regulation by Curcumin. Inflammation (2019) 42(4):1160–9. 10.1007/s10753-019-01010-4 31011925

[7] Freitas A Alves-Filho JC Victoni T Secher T Lemos HP Sônego F. Il-17 Receptor Signaling is Required to Control Polymicrobial Sepsis. J Immunol (2009) 182(12):7846–54. 10.4049/jimmunol.0803039 19494309

[8] Ogiku M Kono H Hara M Tsuchiya M Fujii H. Interleukin-17A Plays a Pivotal Role in Polymicrobial Sepsis According to Studies Using IL-17A Knockout Mice. J Surg Res (2012) 174(1):142–9. 10.1016/j.jss.2010.11.901 21227459

[9] Costa MF de Negreiros CB Bornstein VU Valente RH Mengel J Henriques M. Murine IL-17+ Vγ4 T Lymphocytes Accumulate in the Lungs and Play a Protective Role During Severe Sepsis. BMC Immunol (2015) 16:36. 10.1186/s12865-015-0098-8 26037291 PMC4451961

[10] Ramakrishnan SK Zhang H Ma X Jung I Schwartz AJ Triner D. Intestinal non-Canonical Nfκb Signaling Shapes the Local and Systemic Immune Response. Nat Commun (2019) 10(1):660. 10.1038/s41467-019-08581-8 30737385 PMC6368617

[11] Cua DJ Tato CM. Innate IL-17-producing Cells: The Sentinels of the Immune System. Nat Rev Immunol (2010) 10(7):479–89. 10.1038/nri2800 20559326

[12] McGeachy MJ Cua DJ Gaffen SL. The IL-17 Family of Cytokines in Health and Disease. Immunity (2019) 50(4):892–906. 10.1016/j.immuni.2019.03.021 30995505 PMC6474359

[13] Lai D Tang J Chen L Fan EK Scott MJ Li Y. Group 2 Innate Lymphoid Cells Protect Lung Endothelial Cells From Pyroptosis in Sepsis. Cell Death Dis (2018) 9(3):369. 10.1038/s41419-018-0412-5 29511181 PMC5840374

[14] Huang Y Guo L Qiu J Chen X Hu-Li J Siebenlist U. Il-25-responsive, Lineage-Negative KLRG1(hi) Cells are Multipotential ‘Inflammatory’ Type 2 Innate Lymphoid Cells. Nat Immunol (2015) 16(2):161–9. 10.1038/ni.3078 PMC4297567 25531830

[15] Zhang K Xu X Pasha MA Siebel CW Costello A Haczku A. Cutting Edge: Notch Signaling Promotes the Plasticity of Group-2 Innate Lymphoid Cells. J Immunol (2017) 198(5):1798–803. 10.4049/jimmunol.1601421 PMC5321819 28115527

[16] Koyasu S. Inflammatory ILC2 Cells: Disguising Themselves as Progenitors? Nat Immunol (2015) 16(2):133–4. 10.1038/ni.3080 25594455

[17] Cai T Qiu J Ji Y Li W Ding Z Suo C. IL-17-Producing ST2(+) Group 2 Innate Lymphoid Cells Play a Pathogenic Role in Lung Inflammation. J Allergy Clin Immunol (2019) 143(1):229–244 e9. 10.1016/j.jaci.2018.03.007 29625134 PMC6170730

[18] Cardoso V Chesne J Ribeiro H Garcia-Cassani B Carvalho T Bouchery T. Neuronal Regulation of Type 2 Innate Lymphoid Cells Via Neuromedin U. Nature (2017) 549(7671):277–81. 10.1038/nature23469 PMC5714273 28869974

[19] Wallrapp A Riesenfeld SJ Burkett PR Abdulnour RE Nyman J Dionne D. The Neuropeptide NMU Amplifies ILC2-driven Allergic Lung Inflammation. Nature (2017) 549(7672):351–6. 10.1038/nature24029 PMC5746044 28902842

[20] Nagashima H Mahlakõiv T Shih HY Davis FP Meylan F Huang Y. Neuropeptide CGRP Limits Group 2 Innate Lymphoid Cell Responses and Constrains Type 2 Inflammation. Immunity (2019) 51(4):682–95.e6. 10.1016/j.immuni.2019.06.009 31353223 PMC6801073

[21] Galle-Treger L Suzuki Y Patel N Sankaranarayanan I Aron JL Maazi H. Nicotinic Acetylcholine Receptor Agonist Attenuates ILC2-dependent Airway Hyperreactivity. Nat Commun (2016) 7:13202. 10.1038/ncomms13202 27752043 PMC5071851

[22] Klose CSN Mahlakoiv T Moeller JB Rankin LC Flamar AL Kabata H. The Neuropeptide Neuromedin U Stimulates Innate Lymphoid Cells and Type 2 Inflammation. Nature (2017) 549(7671):282–6. 10.1038/nature23676 PMC6066372 28869965

[23] Piliponsky AM Chen CC Grimbaldeston MA Burns-Guydish SM Hardy J Kalesnikoff J. Mast Cell-Derived TNF can Exacerbate Mortality During Severe Bacterial Infections in C57BL/6-KitW-sh/W-sh Mice. Am J Pathol (2010) 176(2):926–38. 10.2353/ajpath.2010.090342 PMC2808097 20035049

[24] Moro K Ealey KN Kabata H Koyasu S. Isolation and Analysis of Group 2 Innate Lymphoid Cells in Mice. Nat Protoc (2015) 10(5):792–806. 10.1038/nprot.2015.047 25927389

[25] von Moltke J O’Leary CE Barrett NA Kanaoka Y Austen KF Locksley RM. Leukotrienes Provide an NFAT-dependent Signal That Synergizes With IL-33 to Activate ILC2s. J Exp Med (2017) 214(1):27–37. 10.1084/jem.20161274 28011865 PMC5206504

[26] Ribot JC Serre K Silva-Santos B. Developmental and Functional Assays to Study Murine and Human γδ T Cells. Methods Mol Biol (2017) 1514:257–67. 10.1007/978-1-4939-6548-9_18 27787805

[27] Sutton CE Lalor SJ Sweeney CM Brereton CF Lavelle EC Mills KH. Interleukin-1 and IL-23 Induce Innate IL-17 Production From Gammadelta T Cells, Amplifying Th17 Responses and Autoimmunity. Immunity (2009) 31(2):331–41. 10.1016/j.immuni.2009.08.001 19682929

[28] Lopes N McIntyre C Martin S Raverdeau M Sumaria N Kohlgruber AC. Distinct Metabolic Programs Established in the Thymus Control Effector Functions of γδ T Cell Subsets in Tumor Microenvironments. Nat Immunol (2021) 22(2):179–92. 10.1038/s41590-020-00848-3 PMC7610600 33462452

[29] de Jong HK van der Poll T Wiersinga WJ. The Systemic Pro-Inflammatory Response in Sepsis. J Innate Immun (2010) 2(5):422–30. 10.1159/000316286 20530955

[30] Amatya N Garg AV Gaffen SL. Il-17 Signaling: The Yin and the Yang. Trends Immunol (2017) 38(5):310–22. 10.1016/j.it.2017.01.006 PMC5411326 28254169

[31] Loser S Maizels RM. Immunology: The Neuronal Pathway to Mucosal Immunity. Curr Biol (2018) 28(1):R33–6. 10.1016/j.cub.2017.11.025 29316420

[32] Gasse P Riteau N Vacher R Michel ML Fautrel A di Padova F. IL-1 and IL-23 Mediate Early IL-17A Production in Pulmonary Inflammation Leading to Late Fibrosis. PloS One (2011) 6(8):e23185. 10.1371/journal.pone.0023185 21858022 PMC3156735

[33] Guggino G Ciccia F Di Liberto D Lo Pizzo M Ruscitti P Cipriani P. Interleukin (Il)-9/IL-9R Axis Drives γδ T Cells Activation in Psoriatic Arthritis Patients. Clin Exp Immunol (2016) 186(3):277–83. 10.1111/cei.12853 PMC5108067 27543964

[34] Iwakura Y Nakae S Saijo S Ishigame H. The Roles of IL-17A in Inflammatory Immune Responses and Host Defense Against Pathogens. Immunol Rev (2008) 226:57–79. 10.1111/j.1600-065X.2008.00699.x 19161416

[35] O’Connor W Kamanaka M Booth CJ Town T Nakae S Iwakura Y. A Protective Function for Interleukin 17A in T Cell-Mediated Intestinal Inflammation. Nat Immunol (2009) 10(6):603–9. 10.1038/ni.1736 PMC2709990 19448631

[36] Flierl MA Rittirsch D Gao H Hoesel LM Nadeau BA Day DE. Adverse Functions of IL-17A in Experimental Sepsis. FASEB J (2008) 22(7):2198–205. 10.1096/fj.07-105221 18299333

[37] Rendon JL Choudhry MA. Th17 Cells: Critical Mediators of Host Responses to Burn Injury and Sepsis. J Leukoc Biol (2012) 92(3):529–38. 10.1189/jlb.0212083 PMC3427614 22753950

[38] Wynn JL Wilson CS Hawiger J Scumpia PO Marshall AF Liu JH. Targeting IL-17A Attenuates Neonatal Sepsis Mortality Induced by IL-18. Proc Natl Acad Sci USA (2016) 113(19):E2627–35. 10.1073/pnas.1515793113 PMC4868456 27114524

[39] Schüler R Brand A Klebow S Wild J Veras FP Ullmann E. Antagonization of IL-17A Attenuates Skin Inflammation and Vascular Dysfunction In Mouse Models of Psoriasis. J Invest Dermatol (2019) 139(3):638–47. 10.1016/j.jid.2018.09.021 30367871

[40] Yadava K Pattaroni C Sichelstiel AK Trompette A Gollwitzer ES Salami O. Microbiota Promotes Chronic Pulmonary Inflammation by Enhancing Il-17A and Autoantibodies. Am J Respir Crit Care Med (2016) 193(9):975–87. 10.1164/rccm.201504-0779OC 26630356

[41] Chen W Shu Q Fan J. Neural Regulation of Interactions Between Group 2 Innate Lymphoid Cells and Pulmonary Immune Cells. Front Immunol (2020) 11:576929. 10.3389/fimmu.2020.576929 33193374 PMC7658006

[42] Branzk N Gronke K Diefenbach A. Innate Lymphoid Cells, Mediators of Tissue Homeostasis, Adaptation and Disease Tolerance. Immunol Rev (2018) 286(1):86–101. 10.1111/imr.12718 30294961

[43] Sonnenberg GF Artis D. Innate Lymphoid Cells in the Initiation, Regulation and Resolution of Inflammation. Nat Med (2015) 21(7):698–708. 10.1038/nm.3892 26121198 PMC4869856

[44] Kabata H Moro K Koyasu S. The Group 2 Innate Lymphoid Cell (ILC2) Regulatory Network and its Underlying Mechanisms. Immunol Rev (2018) 286(1):37–52. 10.1111/imr.12706 30294963

[45] Moriyama M Furue H Katafuchi T Teranishi H Sato T Kano T. Presynaptic Modulation by Neuromedin U of Sensory Synaptic Transmission in Rat Spinal Dorsal Horn Neurones. J Physiol (2004) 559(Pt 3):707–13. 10.1113/jphysiol.2004.070110 PMC1665182 15297576

[46] Hedrick JA Morse K Shan L Qiao X Pang L Wang S. Identification of a Human Gastrointestinal Tract and Immune System Receptor for the Peptide Neuromedin U. Mol Pharmacol (2000) 58(4):870–5. 10.1124/mol.58.4.870 10999960

[47] Vantourout P Hayday A. Six-of-the-Best: Unique Contributions of γδ T Cells to Immunology. Nat Rev Immunol (2013) 13(2):88–100. 10.1038/nri3384 23348415 PMC3951794

[48] Carding SR Egan PJ. Gammadelta T Cells: Functional Plasticity and Heterogeneity. Nat Rev Immunol (2002) 2(5):336–45. 10.1038/nri797 12033739

[49] Hedges JF Lubick KJ Jutila MA. Gamma Delta T Cells Respond Directly to Pathogen-Associated Molecular Patterns. J Immunol (2005) 174(10):6045–53. 10.4049/jimmunol.174.10.6045 15879098

[50] Welsh KJ Risin SA Actor JK Hunter RL. Immunopathology of Postprimary Tuberculosis: Increased T-regulatory Cells and DEC-205-positive Foamy Macrophages in Cavitary Lesions. Clin Dev Immunol (2011) 2011:307631. 10.1155/2011/307631 21197439 PMC3010642

[51] Uldrich AP Le Nours J Pellicci DG Gherardin NA McPherson KG Lim RT. CD1d-Lipid Antigen Recognition by the γδ Tcr. Nat Immunol (2013) 14(11):1137–45. 10.1038/ni.2713 24076636

[52] Raulet DH Gasser S Gowen BG Deng W Jung H. Regulation of Ligands for the NKG2D Activating Receptor. Annu Rev Immunol (2013) 31:413–41. 10.1146/annurev-immunol-032712-095951 PMC4244079 23298206

[53] Silva-Santos B. γδ Cells Making IL-17. Blood (2011) 118(1):3–5. 10.1182/blood-2011-05-351726 21737606

[54] Ribot JC deBarros A Pang DJ Neves JF Peperzak V Roberts SJ. CD27 is a Thymic Determinant of the Balance Between Interferon-Gamma- and Interleukin 17-Producing Gammadelta T Cell Subsets. Nat Immunol (2009) 10(4):427–36. 10.1038/ni.1717 PMC4167721 19270712

[55] Wu P Wu D Ni C Ye J Chen W Hu G. γδt17 Cells Promote the Accumulation and Expansion of Myeloid-Derived Suppressor Cells in Human Colorectal Cancer. Immunity (2014) 40(5):785–800. 10.1016/j.immuni.2014.03.013 24816404 PMC4716654

[56] Haas JD González FH Schmitz S Chennupati V Föhse L Kremmer E. CCR6 and NK1.1 Distinguish Between IL-17A and IFN-gamma-producing Gammadelta Effector T Cells. Eur J Immunol (2009) 39(12):3488–97. 10.1002/eji.200939922 19830744

[57] Kong X Sun R Chen Y Wei H Tian Z. γδt Cells Drive Myeloid-Derived Suppressor Cell-Mediated CD8+ T Cell Exhaustion in Hepatitis B Virus-Induced Immunotolerance. J Immunol (2014) 193(4):1645–53. 10.4049/jimmunol.1303432 25015833

[58] Zheng J Liu Y Lau YL Tu W. γδ-T Cells: An Unpolished Sword in Human Anti-Infection Immunity. Cell Mol Immunol (2013) 10(1):50–7. 10.1038/cmi.2012.43 PMC4003172 23064104

[59] Papotto PH Ribot JC Silva-Santos B. Il-17+ Gammadelta T Cells as Kick-Starters of Inflammation. Nat Immunol (2017) 18(6):604–11. 10.1038/ni.3726 28518154

[60] Hirsh MI Hashiguchi N Chen Y Yip L Junger WG. Surface Expression of HSP72 by LPS-stimulated Neutrophils Facilitates Gammadeltat Cell-Mediated Killing. Eur J Immunol (2006) 36(3):712–21. 10.1002/eji.200535422 16482515

[61] Chung CS Watkins L Funches A Lomas-Neira J Cioffi WG Ayala A. Deficiency of Gammadelta T Lymphocytes Contributes to Mortality and Immunosuppression in Sepsis. Am J Physiol Regul Integr Comp Physiol (2006) 291(5):R1338–43. 10.1152/ajpregu.00283.2006 PMC1592544 16793935

[62] Enoh VT Lin SH Lin CY Toliver-Kinsky T Murphey ED Varma TK. Mice Depleted of Alphabeta But Not Gammadelta T Cells are Resistant to Mortality Caused by Cecal Ligation and Puncture. Shock (2007) 27(5):507–19. 10.1097/SHK.0b013e31802b5d9f 17438456

[63] Tschöp J Martignoni A Goetzman HS Choi LG Wang Q Noel JG. Gammadelta T Cells Mitigate the Organ Injury and Mortality of Sepsis. J Leukoc Biol (2008) 83(3):581–8. 10.1189/jlb.0707507 PMC2747639 18063696

[64] Xu R Wang R Han G Wang J Chen G Wang L. Complement C5a Regulates IL-17 by Affecting the Crosstalk Between DC and Gammadelta T Cells in CLP-induced Sepsis. Eur J Immunol (2010) 40(4):1079–88. 10.1002/eji.200940015 20140904

[65] Kasten KR Prakash PS Unsinger J Goetzman HS England LG Cave CM. Interleukin-7 (Il-7) Treatment Accelerates Neutrophil Recruitment Through Gamma Delta T-cell Il-17 Production in a Murine Model of Sepsis. Infect Immun (2010) 78(11):4714–22. 10.1128/IAI.00456-10 PMC2976361 20823197

[66] Rice L Orlow D Ceonzo K Stahl GL Tzianabos AO Wada H. Cpg Oligodeoxynucleotide Protection in Polymicrobial Sepsis is Dependent on Interleukin-17. J Infect Dis (2005) 191(8):1368–76. 10.1086/428452 15776385

[67] Alves-Filho JC Sônego F Souto FO Freitas A Verri WA Auxiliadora-Martins M. Interleukin-33 Attenuates Sepsis by Enhancing Neutrophil Influx to the Site of Infection. Nat Med (2010) 16(6):708–12. 10.1038/nm.2156 20473304

[68] Shin SB Lo BC Ghaedi M Scott RW Li Y Messing M. Abortive γδtcr Rearrangements Suggest ILC2s are Derived From T-cell Precursors. Blood Adv (2020) 4(21):5362–72. 10.1182/bloodadvances.2020002758 PMC7656916 33137203

[69] Zhang W Tang T Nie D Wen S Jia C Zhu Z. IL-9 Aggravates the Development of Atherosclerosis in ApoE-/- Mice. Cardiovasc Res (2015) 106(3):453–64. 10.1093/cvr/cvv110 25784693

[70] Mohapatra A Van Dyken SJ Schneider C Nussbaum JC Liang HE Locksley RM. Group 2 Innate Lymphoid Cells Utilize the IRF4-IL-9 Module to Coordinate Epithelial Cell Maintenance of Lung Homeostasis. Mucosal Immunol (2016) 9(1):275–86. 10.1038/mi.2015.59 PMC4698110 26129648

[71] Nascimento DC Melo PH Piñeros AR Ferreira RG Colón DF Donate PB. Il-33 Contributes to Sepsis-Induced Long-Term Immunosuppression by Expanding the Regulatory T Cell Population. Nat Commun (2017) 8:14919. 10.1038/ncomms14919 28374774 PMC5382289

