# Supplementary figures and images for: Neuronal-Activated ILC2s Promote IL-17A Production in Lung γδ T Cells During Sepsis
Source: Front Immunol. 2021 Apr 30;12:670676. doi: 10.3389/fimmu.2021.670676 (PMC8119647; doi:10.3389/fimmu.2021.670676)

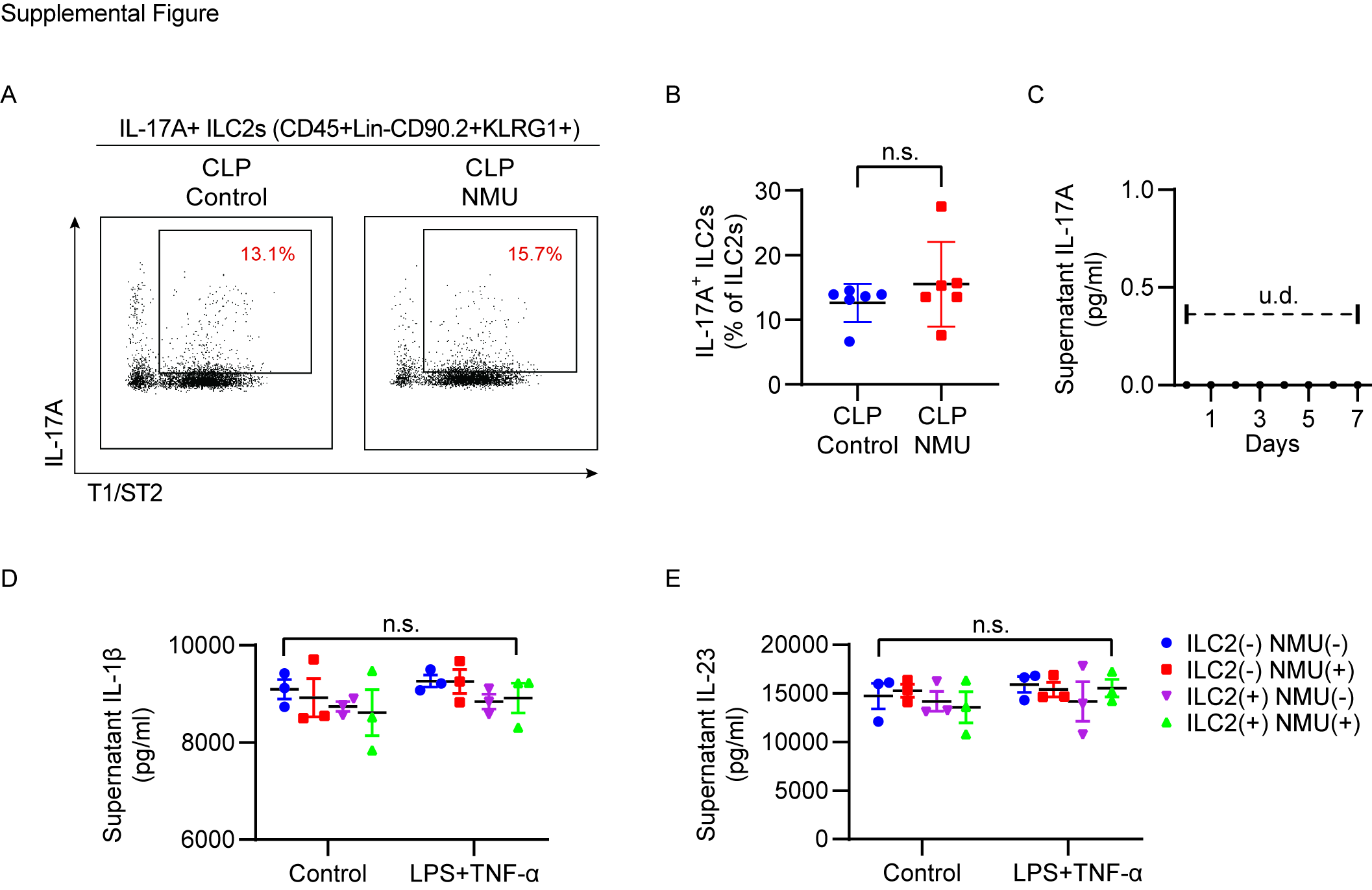

Supplement: Supplementary Figure — (A) Representative flow cytometry plots for IL-17A+ILC2 population within lung live CD45+Lineage-CD90.2+ populations at 24h after CLP. (B) The percentages of IL-17A+ ILC2 population within lung ILC2 population at 24h after CLP (n = 6). (C) ELISA analysis of supernatant IL-17A in ILC2 alone group. ILC2s were treated with NMU (10 µg/ml) (n = 3). (D, E) ELISA analysis of supernatant IL-1β (D) and IL-23 (E) in different groups. ILC2s and γδ T cells were co-cultured for 48h with or without NMU (10 μg/ml). IL-1β (100 ng/ml) and IL-23 (100 ng/ml) were added to polarize IL-17A-producing γδ T cells, LPS (1 μg/ml) plus TNF-α (20 ng/ml) were added to mimic sepsis stimulation (n = 3). All data are mean ± SEM. n.s., not significant, u.d., undetected. One-way ANOVA in (D, E); two-tailed Student’s t-test in (B). [file Image_1.tif]
